# Antidiabetic Potential of the Heme Oxygenase-1 Inducer Curcumin Analogues

**DOI:** 10.1155/2013/918039

**Published:** 2013-09-26

**Authors:** Yong Son, Ju Hwan Lee, Yong-Kwan Cheong, Hun-Taeg Chung, Hyun-Ock Pae

**Affiliations:** ^1^Department of Anesthesiology and Pain Medicine, Wonkwang University School of Medicine, 460 Iksandae-ro, Iksan 570-749, Republic of Korea; ^2^Department of Biological Science, University of Ulsan, 30 Daehack-ro, Ulsan 680-749, Republic of Korea; ^3^Department of Microbiology and Immunology, Wonkwang University School of Medicine, 460 Iksandae-ro, Iksan 570-749, Republic of Korea

## Abstract

Although there is a therapeutic treatment to combat diabetes, the identification of agents that may deal with its more serious aspects is an important medical field for research. Diabetes, which contributes to the risk of cardiovascular disease, is associated with a low-grade chronic inflammation (inflammatory stress), oxidative stress, and endoplasmic reticulum (ER) stress. Because the integration of these stresses is critical to the pathogenesis of diabetes, agents and cellular molecules that can modulate these stress responses are emerging as potential targets for intervention and treatment of diabetic diseases. It has been recognized that heme oxygenase-1 (HO-1) plays an important role in cellular protection. Because HO-1 can reduce oxidative stress, inflammatory stress, and ER stress, in part by exerting antioxidant, anti-inflammatory, and antiapoptotic effects, HO-1 has been suggested to play important roles in pathogenesis of diabetes. In the present review, we will explore our current understanding of the protective mechanisms of HO-1 in diabetes and present some emerging therapeutic options for HO-1 expression in treating diabetic diseases, together with the therapeutic potential of curcumin analogues that have their ability to induce HO-1 expression.

## 1. Introduction

A number of studies have suggested that diabetes mellitus, the hallmark of which consists of elevated plasma glucose, is consistently associated with oxidative stress, a chronic low-grade inflammation (hereafter referred as to “inflammatory stress”), and endoplasmic reticulum (ER) stress [1, 2]. Moreover, it is most likely that oxidative stress, inflammatory stress, and ER stress may interact with each other during pathogenesis of diabetes, and they, as a result, may be amplified to ultimately induce abnormal cell death. Thus, agents and/or cellular defense molecules that can modulate these stress responses are emerging as potential targets for intervention and treatment of diabetes. Numerous experimental studies have confirmed the important role of naturally occurring phytochemicals in prevention and treatment of diabetes, particularly associated with oxidative stress [3]. Of them, the nutritional antioxidant curcumin (Cur) has been highlighted [4]; therefore, we will discuss the therapeutic use of Cur, in the context of oxidative stress-related diseases, together with the underlying mechanisms of its action.

Turmeric is prepared by grinding dried rhizomes of *Curcuma longa*. Traditionally, turmeric has been used as a foodstuff and has been an important component of Indian medicine and traditional Chinese medicine [5]. Cur is one of the active components responsible for the majority of the medicinal properties of turmeric. Cur has been shown to protect against oxidative stress [4–6]. However, the mechanisms of actions involved in the antioxidant-related protective effects of Cur are not fully understood. Cur has been reported to have the capacity to directly quench reactive oxygen species (ROS) that can contribute to oxidative damage [7]. While this property of Cur is known to contribute to its overall protective effects, Cur can also have the capacity to activate or inhibit various cellular signaling pathways and numerous additional regulatory molecules involved in cellular protection against oxidative stress [8]. Interestingly, recent studies have shown that Cur can attenuate cell death caused by oxidative stress, indirectly through induction and/or activation of antioxidant/cytoprotective enzymes, such as heme oxygenase-1 (HO-1) [9].

HO-1, a ubiquitous inducible cellular stress protein, serves a major metabolic function as the rate-limiting step in the oxidative catabolism of heme, leading to formation of equimolar amounts of biliverdin (BV), free iron, and carbon monoxide (CO); the BV formed in this reaction is rapidly converted to bilirubin (BR) by BV reductase [10]. It has become increasingly recognized that HO-1, an inducible enzyme, plays an important role in cellular protection [11]. The protective biological activities conferred by HO-1 include antioxidant, anti-inflammatory, and antiapoptotic properties [10, 11]. By virtue of such protective activities, HO-1 has been suggested to play important roles in pathogenesis of diabetic diseases [12]. In the present review, we will explore our current understanding of the protective mechanisms of HO-1 in diabetes and present some emerging therapeutic options for HO-1 expression in treating diabetic complications, together with the therapeutic potential of Cur analogues.

## 2. Cellular Stresses in Diabetes

A growing body of evidence suggests an early and central role of increased oxidative stress as a causal pathway linking with diabetic diseases [1–3]. Besides oxidative stress, ER stress and inflammatory stress are often present in the patients with diabetes, whereas they are also related to oxidative stress [1, 2]. Although roles for individual processes, such as oxidative stress, inflammatory stress, and ER stress, in diabetes have been recognized in scattered reports, how these processes are interrelated in bringing about diabetes has not been clear. However, these processes are ultimately integrated in the pathogenesis of diabetes. 

### 2.1. Oxidative Stress

ROS, of which production is an unavoidable consequence of aerobic metabolism in animal cells, consist primarily of the various oxygen free radicals, including superoxide anion radical (O_2_^∙−^) and hydroxyl radical (HO^∙^), as well as the potent oxidizing molecules, including hydrogen peroxide (H_2_O_2_). At their high concentrations, ROS can react with many different macromolecules, thereby causing damage to, for example, DNA, proteins, and lipids [13]. ROS, therefore, play a major role in many disease processes. Despite their destructive activity, low/moderate levels of ROS are indispensable in several biochemical processes, including intracellular messaging and defense against microorganisms [14]. Thus, it is necessary for the cells to control the level of ROS tightly to avoid any oxidative injury and not to eliminate them completely. This is supported by the fact that levels of ROS are tightly regulated by cellular antioxidant defense systems including small antioxidant molecules, such as glutathione, and ROS-scavenging enzymes, such as superoxide dismutase (SOD), catalase, and glutathione peroxidase (GPX) [15]. Thus, oxidative stress has been shown to describe a condition in which these cellular antioxidant defense mechanisms are insufficient to inactivate ROS, or excessive ROS are produced, or both. The novel concepts in our understanding of oxidative stress indicate that a perturbed redox circuitry could be strongly linked with the onset of diabetes.

Nicotinamide adenine dinucleotide phosphate (NADP) oxidase that can produce superoxide in response to an inflammatory response in phagocytotic cells or by receptor tyrosine kinase-mediated engagement in nonphagocytotic cells [16] and the mitochondrial respiratory chain mainly at complexes I and III in the mitochondria are believed to be major sites of ROS production under physiological and pathological conditions. In the mitochondria, ROS are spontaneously generated but rapidly converted to nontoxic molecules by cellular antioxidant molecules, such as SOD, catalase, and GPX. However, in a state of chronic nutrient/energy overload or continued exposure to glucose metabolites, the flux of nutrients through the mitochondrial respiratory chain can be increased, thereby enhancing ROS production, presumably because of insufficient capacity of cellular antioxidants, such as SOD, catalase, and GPX, to neutralize excessive ROS, and eventually inducing oxidative stress. ROS have been hypothesized to inhibit the cell signaling of the insulin receptor by blocking the pathway between insulin-receptor substrate 1 (IRS-1) and phosphatidylinositol-4,5-bisphosphate 3-kinase, thereby inducing insulin resistance (IR) [17]. This hypothesis has been supported by the findings demonstrating that animal models of IR and diabetes are characterized by persistently elevated ROS levels [18].

### 2.2. Inflammatory Stress

Inflammation is a response to eliminate the initial cause of cellular injury as well as the necrotic cells and tissues that result from the original insult. The mechanism causing inflammation during the pathogenesis of diabetic diseases is still under investigation. It is most likely that the immune sensors, such as pattern recognition receptors (PRRs) and other pathogen-sensing kinases, may participate in the development of diabetes. Pathogen-associated molecular pattern molecules (PAMPs) are derived from microorganisms and recognized by PRR-bearing cells of the innate immune system. In contrast, damage-associated molecular pattern molecules (DAMPs) are cell-derived and initiate and perpetuate immunity in response to trauma, ischemia, and tissue damage, either in the absence or presence of pathogenic infection [19]. Most PAMPs and DAMPs bind specific PRRs, such as Toll-like receptors (TLRs) [19]. It is most likely that DAMPs released from the necrotic cells and tissues resulting from their exposure to toxic molecules, such as ROS, may trigger inflammatory response. Interestingly, TLR4 has been reported to be activated by saturated free fatty acids (FAs) to generate inflammatory signals in macrophages, endothelial cells (ECs), and adipocytes, ultimately resulting in the production of proinflammatory cytokines, such as (TNF-*α*) and interleukin-1*β* (IL-1*β*), and ROS [20]. The bacterial endotoxin lipopolysaccharide (LPS) is a classical ligand for TLR4 in most cell types. The majority of the biological activity of LPS is contained within a moiety that is acylated with saturated FAs, and removal of these FAs results in complete loss of its ability to activate TLR4, suggesting that there is a degree of similarity in structure among LPS and saturated free FAs. It is well established that elevated levels of proinflammatory cytokines are detected in patients with the IR-associated clinical states [21–23] and in experimental mouse models of diabetes [24]. 

### 2.3. ER Stress

The ER is a highly dynamic organelle responsible for protein folding, maturation, quality control, and trafficking. When the ER becomes stressed due to the accumulation of newly synthesized unfolded proteins, this condition has been referred to as an “ER stress,” and the unfolded protein response (UPR) is activated to increase protein folding capacity and to decrease unfolded protein [25]. If these mechanisms of adaptation are insufficient to recover ER homeostasis, the UPR will induce cell death programs to eliminate the stressed cells, which may contribute to disease states. In animal cells, the UPR is mediated by at least three transmembrane proteins, including inositol-requiring enzyme 1 (IRE1), protein-kinase-RNA-like ER kinase (PERK), and activating transcription factor 6 (ATF6) [25, 26]. Under unstressed conditions, these transmembrane proteins are maintained in an inactive state by binding to the major ER chaperone, immunoglobulin heavy chain binding protein/glucose-regulated protein 78 (BiP), at the side of the ER lumen. During ER stress, BiP is displaced to interact with misfolded luminal proteins, resulting in the release of IRE1, PERK, and ATF6, and subsequently leading to their activation [25, 26]. PERK activation results in phosphorylation of the *α* subunit of eukaryotic translation initiation factor 2 (eIF2*α*) leading to rapid reduction in the initiation of mRNA translation, and thus reducing the load of new proteins in the ER. Phosphorylation of eIF2*α* by PERK allows the translation of activating transcription factor 4 that can induce transcription of genes involved in amino acids synthesis and apoptosis, such as CCAAT/enhancer-binding protein homologous protein (CHOP) [25, 26]. ER calcium depletion, altered glycosylation, nutrient deprivation, oxidative stress, proinflammatory cytokine, DNA damage, or energy perturbation/fluctuations can interrupt the protein folding process and result in ER stress. A study has demonstrated protection against obesity-induced diabetes in mice by overexpression of ER chaperones, while knockdown of chaperones was diabetogenic [27]. In addition, treatment with chemical ER chaperones that alleviated obesity-induced ER stress led to improvement in insulin sensitivity [27]. 

## 3. HO-1 and Therapeutic Potentials

**Figure 1:**
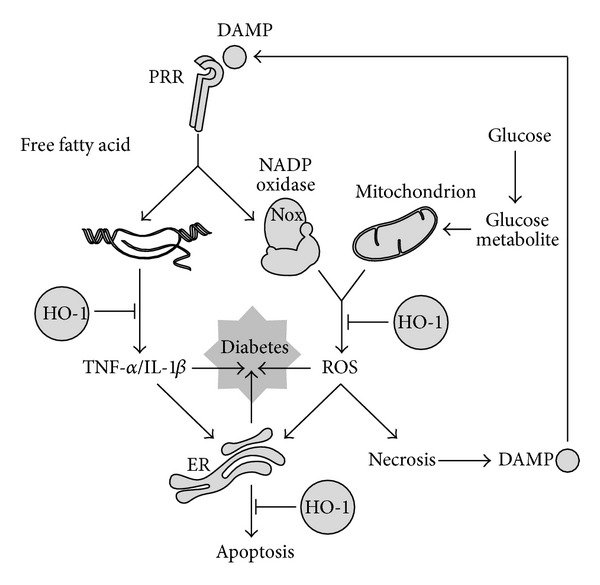
Therapeutic targets of HO-1 during pathogenesis of diabetes. Cells in a tissue may be exposed to oxidative stress generated mainly by mitochondria and NADP oxidase complex, inflammatory stress initiated probably by DAMP-PRR engagement, and ER stress triggered by inflammatory and oxidative stresses, and these stresses, when prolonged, may be amplified and integrated. The integration of amplified stress responses may cause one or more of diabetic complications. HO-1 expression may reduce oxidative stress, inflammatory stress, and ER stress, thereby exerting therapeutic actions.

Although HO-1 is known initially for its role in heme catabolism, HO-1 has become increasingly recognized that HO-1 expression exerts a major role in cellular defense mechanisms [10, 11]. The protective activities conferred by HO-1 expression include its antioxidant, anti-inflammatory, and antiapoptotic properties [10, 11]. These protective effects of HO-1 are dependent on the generation of its enzymatic reaction products (i.e., CO, BV/BR). There is ample evidence that HO-1, in particular, can protect against diabetics (Figure 1) [12]. 

### 3.1. HO-1 against Oxidative Stress

The antioxidant effect of HO-1 has been highlighted in HO-1-knockout mice [27]. As compared with wild-type mice, the liver from HO-1-knockout mice show higher levels of oxidized proteins and lipid peroxidation. Moreover, peritoneal macrophages from HO-1-knockout mice, as compared with wild-type controls, exhibit increased levels of ROS [28]. Similarly, cells from the human case of HO-1 deficiency showed increased sensitivity to oxidative injury [29]. HO-1, therefore, plays a role to counteract oxidative stress, being upregulated during oxidative stress. The specific mechanisms by which HO-1 can mediate antioxidant effect are not clear, but BV and BR, a byproduct generated during the heme catabolism, have been suggested as potential antioxidants. In fact, addition of BR to the culture medium was reported to markedly reduce the cytotoxicity produced by oxidants [10, 11]. Similarly, HO-1 expression by heme increased the resistance against oxidative cell injury; notably, this protective effect occurred only in cells that were actively producing BR [30]. It is important to note that up-regulation of HO-1 is often associated with increased ferritin [31], which sequesters redox-active iron, a toxic byproduct of heme degradation [10, 11]. 

### 3.2. HO-1 against Inflammatory Stress

The anti-inflammatory effect of HO-1 has been also highlighted in HO-1-knockout mice [27]. As compared with wild-type mice, HO-1-knockout mice exhibited hallmarks of a progressive chronic inflammatory state. Peritoneal macrophages from HO-1-knockout mice, as compared with wild-type mice, exhibited increased proinflammatory cytokines [28]. Similarly, a case of human HO-1 deficiency also exhibited hallmarks of a proinflammatory state [29]. The specific mechanisms by which HO-1 can mediate anti-inflammatory effect are not clear, but CO has been suggested as a potential mediator. Studies have shown that administration of CO inhibited the production of LPS-induced proinflammatory cytokines, such as TNF-*α* and IL-1*β* [30], and increased LPS-induced expression of the anti-inflammatory cytokine IL-10 [31]. Several possible mechanisms have been postulated to explain the anti-inflammatory action of CO. CO modulated mitogen-activated protein kinase (MAPK) pathways, including p38 MAPK and c-jun *N*-terminal kinase pathways [10, 11]. CO causes a general down-regulation of proinflammatory cytokine production through p38 MAPK-dependent pathways and nuclear factor-*κ*B inactivation [30]. 

### 3.3. HO-1 against ER Stress

Molecules involved in ER stress response have two opposing functions; adaptive or proapoptotic. ER stress-responsive molecules have an adaptive function in cells that are exposed to mild and transient stresses, whereas these molecules have a proapoptotic function in cells exposed to severe and chronic stress. A study has shown that HO-1 expression was induced in response to ER stress-inducing chemicals, such as thapsigargin, homocysteine and tunicamycin, in smooth muscle cells (SMCs) [32]. Interestingly, exogenous application of the HO-1 byproduct CO inhibited apoptosis induced by ER stress-inducing agents in SMCs, which was associated with the downregulated expression of the proapoptotic proteins. In human endothelial cells, HO-1/CO system also inhibited ER stress-induced apoptosis *via* p38 MAPK-dependent inhibition of the proapoptotic CHOP expression [26]. These studies suggest that HO-1/CO can confer cytoprotection against apoptotic signals originating from ER stress-responsive molecules. 

## 4. HO-1 Inducers and Therapeutic Potentials

Many phytochemicals, which have reported antioxidant and anti-inflammatory properties, could be explored for their potential to reverse oxidative stress, inflammatory stress, and ER stress, which may be finally useful for management of diabetes. HO-1 has been shown to protect against cellular stress-associated physiological disorders on the basis of its rapid up-regulation under various stress conditions and potent physiological regulating properties. Therefore, HO-1 expression has been suggested to have a general adaptive response and enhanced resistance to various stresses [10, 11]. In this regard, pharmacological expression of HO-1 may be a novel therapeutic intervention for diabetes.

### 4.1. HO-1 Expression

Targeted modulation of HO-1 expression for potential therapeutic interventions requires detailed knowledge of the mechanisms that regulate HO-1 gene expression. The nuclear factor-erythroid 2-related factor 2 (Nrf2) is recognized as a major contributor to the up-regulation of multiple antioxidant defense system in response to various phytochemicals. Nrf2 binds to the antioxidant-responsive element (ARE) or the electrophile-responsive element [10, 11]. ARE has been detected in the promoter or upstream promoter regions of the genes encoding phase II antioxidant enzymes including glutathione *S*-transferase subunits, glutamate-cysteine ligase catalytic and glutamate-cysteine ligase modifier subunits, the thioredoxin and peroxiredoxin families, and NAD(P)H:quinone oxidoreductase [10, 11]. HO-1 is upregulated *via* activation of the Nrf2-ARE pathway. Nrf2 activation is mainly controlled by the cytosolic inhibitor Kelch-like enoyl-CoA-hydratase-associated protein1 (Keap1) [10, 11]. Under normal conditions, Nrf2 is anchored in the cytoplasm through binding to Keap1, which, in turn, facilitates the ubiquitination and subsequent proteolysis of Nrf2. Such sequestration and degradation of Nrf2 in the cytoplasm are mechanisms for the repressive effects of Keap1 on Nrf2. Disruption of the Nrf2-Keap1 complex can result from modification of critical cysteines of Keap1. Numerous stimuli cause disruption of the Nrf2-Keap1 complex *via* modulation of these critical cysteines, which permits subsequent nuclear translocation of free Nrf2 [10, 11]. 

### 4.2. Cur Analogues as HO-1 Inducers

**Figure 2:**
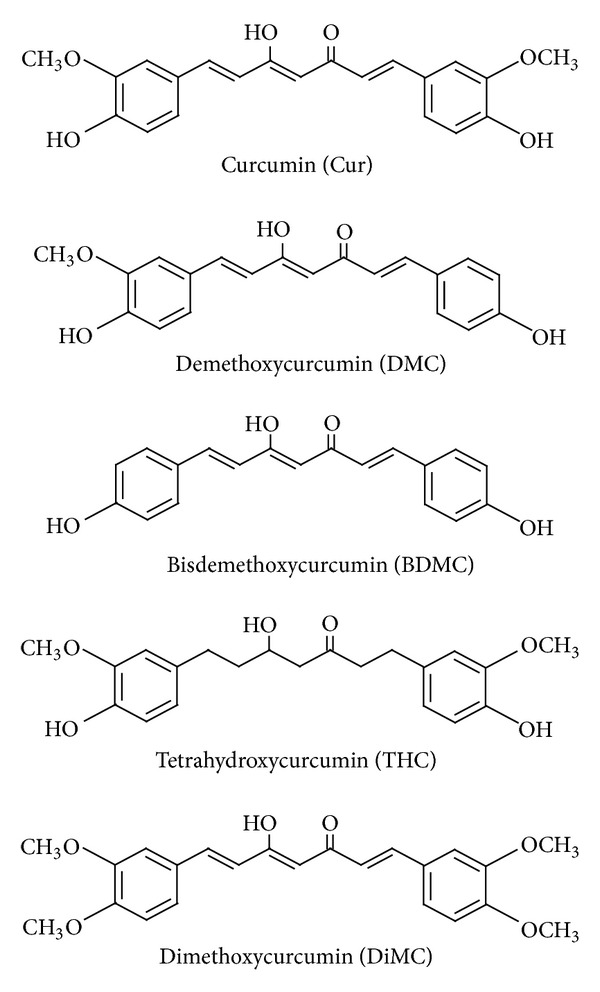
Chemical structures of Cur analogues.

Curcuminoids are the active components responsible for the majority of the medicinal properties of turmeric, and there are 3 naturally occurring curcuminoids: Cur, demethoxycurcumin (DMC), and *bis*-demethoxycurcumin (BDMC). Tetrahydrocurcumin (THC) is one of the major metabolites of Cur, and dimethoxycurcumin (DiMC) is one of synthesized Cur derivatives. The chemical structures of Cur analogues are shown in Figure 2. While Cur contains two methoxyl groups at its *ortho*-position, DMC contains only one and BDMC contains none. In comparison with Cur, DiMC contains additional two methoxyl groups instead of two hydroxyl groups, and THC, like Cur, contains two methoxyl groups and two hydroxyl groups but lacks conjugated double bonds in the central seven-carbon chain. Cur has been first reported to induce *in vitro* HO-1 expression through Keap1-Nrf2/ARE pathway in renal epithelial cells [33], which was further confirmed in rat vascular SMCs [34]. It has been shown that the *α*,*β*-unsaturated carbonyl group may be an important structure of curcuminoids, because THC, lacking this functional group, was virtually inactive in inducing HO-1 expression [34]. In fact, compounds carrying this reactive group have been reported to induce HO-1 expression through activation of Nrf2 nuclear translocation [35]. It has been noted that three naturally occurring curcuminoids vary in their ability to induce HO-1 expression in human endothelial cells [36]. The levels of HO-1 expression were found to be highest with Cur, followed by DMC and BDMC. Considering that the main difference among the three curcuminoids is the number of methoxyl groups (none for BDMC, one for DMC, and two for curcumin), the presence of methoxyl groups in the *ortho*-position on the aromatic ring has been suggested to be essential to enhance HO-1 expression [36], and this finding may be useful in designing more efficacious HO-1 inducers. Cur is rapidly metabolized *in vivo* into THC and other reduced forms [37]. Moreover, HO-1-inducing property of Cur is lost when it is reduced to THC [34, 35]. Thus, there is a need to develop Cur analogues with higher metabolic stability than the original Cur. DiMC, one of several synthetic Cur analogues, was reported to have increased metabolic stability in comparison with Cur [38], and, similar to Cur, induced HO-1 expression *via* Nrf2 activation in RAW264.7 macrophages [35]. Recently, a novel water soluble Cur derivative (NCD) has been developed to overcome low *in vivo* bioavailability of Cur and to evaluate its therapeutic effects in rats with diabetes mellitus [39]. Administration of oral NCD or pure Cur to diabetic rats significantly decreased blood glucose levels and increased the plasma insulin, as compared with the diabetic group, and NCD was more effective in such effects than Cur. Oral NCD did not change the plasma glucose levels in the control group, while it significantly increased the plasma insulin in the control group. Interestingly, treatment of diabetic rats receiving oral NCD with the HO-1 inhibitor zinc protoporphyrin resulted in a significant increase in the plasma glucose level and a significant decrease in insulin levels, when compared with the diabetic group receiving oral NCD only. Administration of oral NCD or pure Cur significantly increased the HO-1 expression level in the pancreatic tissues of the diabetic group, as compared with controls. Thus, it was suggested that the hypoglycemic action of Cur might be mediated through HO-1 expression. 

## 5. Conclusions

There may be the integration of oxidative stress, inflammatory stress, and ER stress in the pathogenesis of diabetes. Depending on the cell type and physiological process, either oxidative stress, inflammatory stress, or ER stress may be more prominent or upstream of the others. However, these signaling pathways may interact and be ultimately integrated in the pathogenesis of diabetes. Given the integration of oxidative stress, inflammatory stress, and ER stress, targeting only one of them may not be effective in controlling disease pathogenesis. As abovementioned, HO-1 has its potential ability to modulate oxidative stress, inflammatory stress, and ER stress, and this has generated immense interest in HO-1 as a therapeutic target (Figure 1). Metalloporphyrins, such as CoPP and hemin, which are prototypical inducers of HO-1 and are commonly used in experimental cell culture and animal models, do not seem to be applicable for clinical interventions, because they lack cell-specificity and are severely toxic when it is used for long periods of time. Naturally occurring phytochemicals ameliorate the risk factors that lead to the development of diabetes, but the mechanisms of their actions remain to be established. Besides their capacity to directly quench ROS, some of them, such as Cur, reduce the incidence of diabetes *via* HO-1 expression [3–9], which allows them to be considered as HO-1 inducers that may provide an alternative strategy for controlling the initiation and progression of diabetic diseases. However, their introduction into the clinical setting may be hindered largely by their poor solubility, rapid metabolism, or a combination of both, ultimately resulting in low therapeutic concentrations at the target site. To overcome the bioavailability, advanced drug delivery systems, designed to provide localized or targeted delivery of these agents, may provide a more viable therapeutic option in the treatment of diabetes.
